# Supporting Mathematical Argumentation and Proof Skills: Comparing the Effectiveness of a Sequential and a Concurrent Instructional Approach to Support Resource-Based Cognitive Skills

**DOI:** 10.3389/fpsyg.2020.572165

**Published:** 2021-01-21

**Authors:** Daniel Sommerhoff, Ingo Kollar, Stefan Ufer

**Affiliations:** ^1^Department of Mathematics, LMU Munich, Munich, Germany; ^2^Augsburg University, Augsburg, Germany

**Keywords:** instructional design, mathematics, mathematics education, whole-task learning, mathematical proof, higher education, resource-based cognitive skills, argumentation

## Abstract

An increasing number of learning goals refer to the acquisition of cognitive skills that can be described as ‘resource-based,’ as they require the availability, coordination, and integration of multiple underlying resources such as skills and knowledge facets. However, research on the support of cognitive skills rarely takes this resource-based nature explicitly into account. This is mirrored in prior research on mathematical argumentation and proof skills: Although repeatedly highlighted as resource-based, for example relying on mathematical topic knowledge, methodological knowledge, mathematical strategic knowledge, and problem-solving skills, little evidence exists on how to support mathematical argumentation and proof skills based on its resources. To address this gap, a quasi-experimental intervention study with undergraduate mathematics students examined the effectiveness of different approaches to support both mathematical argumentation and proof skills and four of its resources. Based on the part-/whole-task debate from instructional design, two approaches were implemented during students’ work on proof construction tasks: (i) a *sequential approach* focusing and supporting each resource of mathematical argumentation and proof skills sequentially after each other and (ii) a *concurrent approach* focusing and supporting multiple resources concurrently. Empirical analyses show pronounced effects of both approaches regarding the resources underlying mathematical argumentation and proof skills. However, the effects of both approaches are mostly comparable, and only mathematical strategic knowledge benefits significantly more from the concurrent approach. Regarding mathematical argumentation and proof skills, short-term effects of both approaches are at best mixed and show differing effects based on prior attainment, possibly indicating an expertise reversal effect of the relatively short intervention. Data suggests that students with low prior attainment benefited most from the intervention, specifically from the concurrent approach. A supplementary qualitative analysis showcases how supporting multiple resources concurrently alongside mathematical argumentation and proof skills can lead to a synergistic integration of these during proof construction and can be beneficial yet demanding for students. Although results require further empirical underpinning, both approaches appear promising to support the resources underlying mathematical argumentation and proof skills and likely also show positive long-term effects on mathematical argumentation and proof skills, especially for initially weaker students.

## Introduction

Today, educators in formal and informal learning settings deal with increasingly complex skills as learning goals, such as argumentation or complex problem solving (e.g., National Research Council, 2012; Osborne, 2013; Greiff et al., 2014), which require the availability, coordination, and integration of multiple underlying cognitive resources.

Research from educational psychology focusing on the support of complex skills has long been examining part- and whole-task approaches for learning (e.g., Naylor and Briggs, 1963; Anderson, 1968; Lim et al., 2009). Here, *part-task approaches* focus on the acquisition of individual part tasks or steps within a larger task to later integrate these into the whole task, whereas *whole-task approaches* focus on the immediate acquisition of the larger, entire task. Cumulative evidence from corresponding research of the last decades generally points to a higher effectiveness of whole-task approaches to support complex skills (e.g., van Merriënboer and Kester, 2007; Melo and Miranda, 2016).

Respective research has focused on different parts of larger, complex tasks, which can be decomposed into a number of discrete subtasks, and how those can be learned and transferred to the overall task. It did not focus on different (dispositional) resources possibly required for a specific skill. Still, research from (educational) psychology and mathematics education (e.g., Koeppen et al., 2008; Schoenfeld, 2010; Blömeke et al., 2015) has increasingly stressed the fact that many skills currently focused as educational goals, such as mathematical argumentation and proof skills, rely heavily on several underlying resources that need to be coordinated and integrated to solve problems or successfully meet situations requiring the skill. Researchers increasingly acknowledge that these skills should be conceptualized as *resource-based cognitive skills*. However, these underlying resources are rarely considered in the design of learning environments. Although the idea of supporting a resource-based cognitive skill by “simply” supporting its resources and their application is compelling, an instructional dilemma arises: To foster the overarching skill, is it favorable to focus on each resource and the support of its acquisition sequentially? Or should the focus rather be on all resources and their joint application, concurrently? Both approaches appear to have advantages: The first approach benefits from a higher decomposition and instructional clarity as all resources are addressed individually, yet also requires the later transfer from the individual resources to the overall skill. In contrast, the second approach may overwhelm students with the resource-based cognitive skill and its underlying resources all at once, yet allows an integrated learning of the resources in authentic settings that support the integration of the resources and already trains their concurrent application within mathematical argumentation and proof tasks.

**FIGURE 1:**
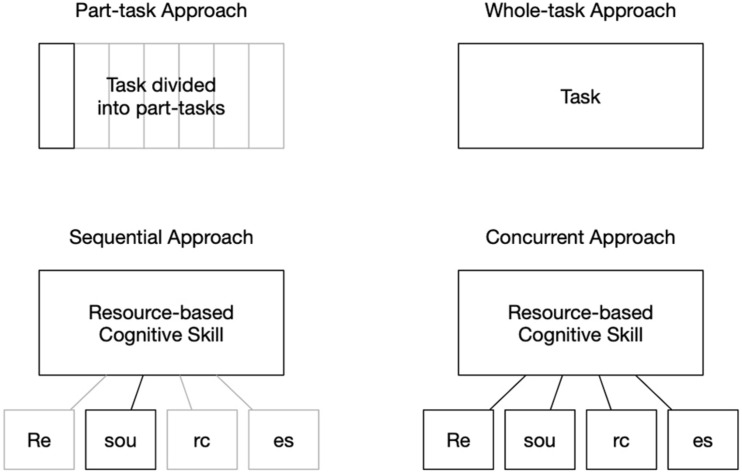
Structural equivalence between the part-/whole-task debate (upper part) and the sequential and concurrent approach to support a resource-based cognitive skill and its resources (lower part).

The dilemma mirrors the part-/whole-task debate (see Figure 1), as (i) supporting each resource underlying a resource-based cognitive skill *sequentially* is analog to the part-task approach, whereas (ii) supporting the resources *concurrently* is analog to the whole-task approach. However, the resources underlying a resource-based cognitive skill go beyond individual steps or subtasks, may have to be purposefully applied within multiple steps, and require more than a sequential enchainment as compared to the individual part tasks. Thus, the transfer of the central tenet from the part-/whole-task debate, that whole-task learning is generally more effective for complex skills, appears questionable and has yet to be investigated thoroughly. In particular, it is generally unclear how effective both approaches for supporting the resources are regarding a complex cognitive skill such as mathematical argumentation and proof skills and whether any learning gains on the resources can be instantly transferred or used for mathematical argumentation and proof.

**FIGURE 2:**
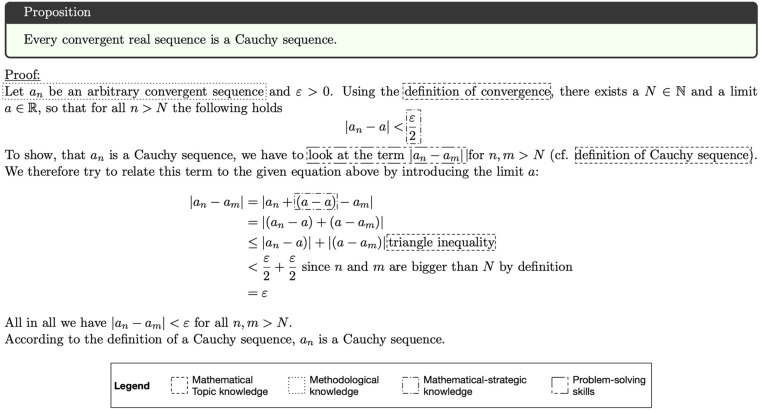
Mathematical proof construction task including solution and exemplary highlights for required resources.

Over the last decades, increasing evidence suggests that *mathematical argumentation and proof skills* should be considered as a resource-based cognitive skill. For example, *mathematical topic knowledge*, *methodological knowledge*, or *problem-solving skills* have been proposed by prior research as underlying resources (e.g., Schoenfeld, 1985; Heinze and Reiss, 2003; Ufer et al., 2008; Chinnappan et al., 2012), for example needed in common proof construction tasks (see Figure 2).

The present contribution addresses the question how mathematical argumentation and proof skills, as a prototype of a *resource-based cognitive skill*, as well as its underlying resources can be effectively supported. We therefore contrast two (resource-based) instructional approaches to support the development of mathematical argumentation and proof skills: a *sequential approach*, focusing and supporting each resource individually one after the other, and a *concurrent approach*, focusing and supporting multiple resources concurrently. We compare students’ learning outcomes in both approaches, regarding both the resources and overall argumentation and proof skills, to give first insights into the effects of both approaches and their feasibility in the context of mathematical argumentation and proof skills and more generally.

## Theoretical Background

### Instructional Approaches for Complex Skills

The idea that instructional strategies to support the learning of less complex skills may differ from those to support more complex skills has been raised repeatedly by educators and prior research (e.g., Branch and Merrill, 2011). Yet, the idea entails serious intricacies, starting with the notion of *skill complexity*, which is ill-defined.

Naylor and Briggs (1963) gave a seminal account of *task difficulty*, differentiating two independent dimensions: *task complexity*, accounting for the individual complexity of the subtasks, and *task organization*, describing the demands posed by the interrelationship between the various subtasks and their integration into the whole task. Their experimental study (Naylor and Briggs, 1963) suggests that tasks with a high subtask complexity but low task organization benefit from part-task training. That is, individual subtasks are trained and afterward connected using different sequencing strategies. In contrast, tasks with low subtask complexity but high task organization benefit from whole-task training, as combining individually learned subtasks is more complex for these tasks. Further, tasks that require not only enchaining but also integrating several subtasks can be more effectively taught using whole-task approaches (Naylor and Briggs, 1963).

Subsequent research contrived plausible theoretical arguments and empirical evidence for both approaches: Arguments for the part-task approach are mostly based on classical learning theories from psychology research like Adaptive Control of Thought (ACT; Anderson, 1996) that assume the decomposability of complex skills into less complex part skills (Anderson, 2002). Based on these assumptions, for example, multiple computer-based approaches like cognitive tutors (Anderson et al., 1995) were developed to support mathematical skills and at least partially proven to be successful. This atomistic approach has been challenged by sociocultural and situated conceptions of learning that highlight the situatedness of learning (e.g., Brown et al., 1989; Lave and Wenger, 1991). The superior effectiveness of whole-task approaches also gained empirical support by evidence pointing to difficulties associated with attempts to transfer and integrate part tasks to the whole task (see Anderson et al., 1996 for a critical discussion).

Several studies and reviews (e.g., Lim et al., 2009; Melo and Miranda, 2015; see van Merriënboer and Kester, 2007 for an overview) document the advantages of whole-task learning for a broad range of learning goals. For example, a meta-analysis on the effects of four-component instructional design (4C/ID) learning environments on school students’ learning (Melo and Miranda, 2016) revealed high positive effects on reproduction (*d* = 0.70) and transfer (*d* = 0.65). Today, many educational theories assume that learning is evoked and supported best by rich, meaningful tasks (van Merriënboer, 2002), which are hard to achieve by focusing on an atomistic approach dissecting whole tasks.

However, empirical studies highlight that, in some situations, the benefits of learning the resources separately may be higher than the challenges of integrating and coordinating the tools in the complex goal task (So et al., 2013) and that additional research may identify which aspects of a skill influence how effective different learning approaches are (Lim et al., 2009; Wickens et al., 2013). For example, Wickens et al. (2013) were able to show that the effectiveness of part-task training depends not only on task difficulty but that the approach for segmenting the whole task into part tasks plays a decisive role. Here, segmenting into parts that have to be used concurrently in the whole task showed a particularly negative effect on the transfer of part-task learning gains to the whole task. Although not prominent in the analysis by Wickens et al. (2013), another aspect discussed repeatedly is prior knowledge or attainment (Salden et al., 2006), as with low prior attainment, both the part tasks and their integration have to be learned.

Still, what has been described as a complex skill in earlier research (c.f., Gagné and Merrill, 1990; van Merriënboer, 1997) seems quite incomparable to skills like argumentation. For example, creating spreadsheets for monthly sales figures (Merrill, 2002) or handling a mechanic excavator (So et al., 2013) cannot be seen as equivalent to argumentation skills, since here not only the integration of several subtasks or subskills in the sense of manual skills, operations, or activities is required but also an integration of various resources underlying the skill, which have to be monitored, coordinated, and regulated. Further, the resources have to be utilized in different ways (for example, when analyzing the task, when creating a plan to solve the task, when solving the task, and when validating the solution), cannot be sequentially enchained, and have to be used concurrently, interacting with each other. It is thus unclear if and how according research can be transferred to more complex cognitive skills and their resources.

### Resource-Based Cognitive Skills

Cognitive skills are often conceptualized in the sense of Koeppen et al. (2008) as latent cognitive dispositions underlying a person’s performance in a range of specified situations. For example, mathematical argumentation and proof skills refer to the cognitive disposition necessary to handle proof-related situations and activities (e.g., Mejía-Ramos and Inglis, 2009). Such situations may ask an individual to construct a valid mathematical proof for a claim or to read a purported proof and judge its correctness. However, judging a person’s success in handling these situations is not straightforward but depends on certain norms to evaluate success. Although norms and values regarding mathematical proofs are generally seen as quite consistent (e.g., Heinze and Reiss, 2003; Dawkins and Weber, 2016), research has still repeatedly shown that they vary to a certain extent (e.g., Inglis et al., 2013; Andersen, 2018) and should be regarded as a social construct that varies depending on the community (e.g., Sommerhoff and Ufer, 2019; see *Method* for a more specific operationalization in the context of this study).

Generally, cognitive skills are not conceived as monolithic, indecomposable latent constructs. Several theoretical, as well as empirical, accounts underline that cognitive skills may heavily require multiple, correlated but potentially independent underlying resources. For example, Shulman (1987) discusses several knowledge facets (e.g., content knowledge, pedagogical knowledge), as underlying teaching skills and, for example, also problem-solving skills are assumed to have underlying resources such as heuristics (e.g., Schoenfeld, 1985; Abel, 2003). A similar conception can be found in vocational education, where Mulder et al. (2009) speak of an “integrated set of capabilities consisting of clusters of knowledge, skills, and attitudes.” The theoretical discussion and framework by Blömeke et al. (2015) integrate these ideas and conceptions, emphasizing the relations between the resources underlying the resource-based cognitive skill and task performance.

This conception of cognitive skills creates a situation that is structurally equivalent to the part-/whole-task debate (see Figure 1). Here, students’ resource-based cognitive skill (e.g., mathematical argumentation and proof skills) can be regarded as analog to the ability to solve whole tasks, whereas the different resources underlying the resource-based cognitive skill (e.g., mathematical topic knowledge) are analog to the ability to solve the part tasks. This analogy substantially extends the part-/whole-task debate, bringing up the question whether the results from the part-/whole-task debate can be transferred to resource-based cognitive skills. Here, the primary question will be, if (i) the resource-based cognitive task can be effectively supported by supporting the resources and (ii) the resources should be supported sequentially one after the other (similar to learning individual part tasks) or whether a concurrent approach (which allows to acquire the resources in a more integrated manner) is more effective. The answers to these questions are highly relevant for the teaching and learning of any resource-based cognitive skill.

Unfortunately, results from prior research (e.g., Salden et al., 2006; Lim et al., 2009) suggest that there might not be *one* answer to this question but that various other aspects, such as students’ prior attainment, might cause differential effects. For example, in an intervention study, Lim et al. (2009) were able to show significant main and interaction effects regarding low vs. high prior attainment and part- vs. whole-task learning for some of their posttest measures, while other measures did not show these effects.

### Mathematical Argumentation and Proof Skills and Its Underlying Resources

Mathematics educators and educational psychologists widely agree that mathematical argumentation and proof skills can be seen as a resource-based cognitive skill (e.g., Schoenfeld, 1985; Ufer et al., 2008; Chinnappan et al., 2012). For example (see also Figure 2), students faced with a mathematical proof task need mathematical topic knowledge to identify the mathematical objects within the task and unpack their definitions and meanings. Further, problem-solving skills may be needed to guide students’ search for a solution and to purposefully apply heuristics to construct a proof.

Several resources of mathematical argumentation and proof skills have been proposed over the last decades (see Sommerhoff et al., 2015 for a review): They have been partly derived from models for more general skills like problem-solving (resources, heuristics, control, belief systems; Schoenfeld, 1985) or self-regulated learning (domain-specific knowledge base, heuristic methods, metaknowledge, self-regulatory skills, beliefs; De Corte et al., 2000) or have been proposed by qualitative studies (mathematical strategic knowledge; Weber, 2001). Moreover, multiple resources have been partially empirically validated (e.g., Ufer et al., 2008; Chinnappan et al., 2012) and shown to account for a large share of students’ variance in mathematical argumentation skills [41.6% explained variance in Ufer et al. (2008) by basic knowledge and problem-solving skills; 72.6% explained variance in Chinnappan et al. (2012) by content knowledge, problem-solving skills, and reasoning skills]. Although quite some research indicates various possible resources of mathematical argumentation skills via theoretical analyses, qualitative analyses, or correlational research, currently no concluding list of such resources, no ranking of their importance, and mostly not even causal evidence justifying their status exist.

Still, based on various frameworks and findings, the following four resources appear to represent important cognitive resources for students’ mathematical argumentation and proof skills:

#### Mathematical Topic Knowledge

One of the most fundamental and best-researched resources is *mathematical topic knowledge (MTK)*. Following widely accepted conceptions (e.g., Hiebert, 1986; Anderson, 1996; Star and Stylianides, 2013), it entails two facets, namely, conceptual mathematical topic knowledge, that is, a network of knowledge about mathematical facts, theorems, objects, and their properties, as well as procedural mathematical topic knowledge, that is, partly tacit knowledge, exercised in the accomplishment of a task (Hiebert and Lefevre, 1986). Both were shown to have a substantial impact on students’ mathematical argumentation and proof skills (Ufer et al., 2008; Chinnappan et al., 2012), matching more general research findings on scientific reasoning (e.g., Schunn and Anderson, 1999; Kuhn, 2002) from psychology.

#### Methodological Knowledge

Meta-knowledge on mathematical argumentation and proof, also called *methodological knowledge (MK)* (Heinze and Reiss, 2003; Ufer et al., 2009; Sommerhoff and Ufer, 2019), is considered another important resource underlying mathematical argumentation and proof skills. It comprises knowledge about acceptance criteria for mathematical proofs (e.g., the rejection of circular reasoning or the need for an explicit reference to an underlying theoretical background) as well as knowledge about different types of proofs, both of which appear particularly essential for constructing and validating proofs.

#### Mathematical Strategic Knowledge

In a qualitative study with mathematics students from different academic levels, Weber (2001) observed that mathematical topic knowledge alone is not sufficient to successfully construct proofs. Students were often unable to identify concepts or methods necessary for a task or had problems applying them purposefully. For example, students could not purposefully apply their (available) knowledge about the fundamental theorem on homomorphisms, as they did not recognize the theorem as purposeful in the specific situation, although the given task included multiple cues implying its usefulness. Data from several other studies (e.g., Reiss and Heinze, 2004; Selden and Selden, 2013) support this finding, implying students’ need for *mathematical strategic knowledge (MSK)*, that is, domain-specific knowledge linking specific cues and hints within mathematical tasks with the mathematical methods and concepts that can be useful for solving the respective task (Weber, 2001). In the broader context of research, mathematical strategic knowledge can be seen as a domain-specific version of general problem-solving heuristics.

In contrast to methodological knowledge, which relates to meta-knowledge about norms and values in the context of mathematical proofs and different types of proofs, mathematical strategic knowledge relates to specific knowledge about how to approach a specific task and discovering cues for such approaches. In particular, similar to the observations by Weber (2001), students may have methodological knowledge about proofs and thus know what the desired proof should look like in terms of its acceptance and features but may still be unable to construct the proof, as they do not know how to approach the given task, implying a need for mathematical strategic knowledge beyond methodological knowledge.

#### Problem-Solving Skills

Next to these three domain-specific resources, problem-solving skills refer to the cognitive disposition to succeed in various problem situations, that is, situations in which an undesired initial state has to be transformed into a goal state, yet the needed operation to achieve this is not at hand (e.g., Dörner, 1979; Mayer and Wittrock, 2006). The specific relation of mathematical proof-construction skills and problem-solving skills as well as the respective processes has been repeatedly discussed (e.g., Mamona-Downs and Downs, 2005; Weber, 2005), resulting in the identification of differences and similarities, and is still a matter of debate. However, based on the definition of problems (e.g., Schoenfeld, 1985) as non-routine tasks for which a learner has no immediate solution strategy, mathematical proofs have often been conceptualized as problems (e.g., Weber, 2005). The construction of a proof can thus be seen as a multistep problem-solving process that, if successful, generates a deductive chain of arguments as a solution for the problem (e.g., Weber, 2005; Heinze et al., 2008). Despite differences between problem solving and proof construction and the fact that today content knowledge is seen as a more important resource, it thus appears plausible that (*general) problem solving skills (PSS)* are a resource for mathematical argumentation and proof skills, which has been underlined repeatedly by prior research (e.g., Polya, 1945; Schoenfeld, 1985; Reiss and Renkl, 2002) and also partially quantitatively underpinned by studies on secondary school students’ geometry proof skills (Ufer et al., 2008; Chinnappan et al., 2012). Simultaneously, the use of problem-solving heuristics, that is, strategies or rules-of-thumb for problem-solving processes, have also been proposed as important for mathematical argumentation and proof skills. These are mostly conceptualized in a way that they are employed when solving a problem and accordingly represent an important resource for problem-solving skills themselves (e.g., Schoenfeld, 1985; Abel, 2003).

Prior research has generally underlined the importance of these four cognitive resources for students’ mathematical argumentation and proof skills. In particular, their importance is supported by quantitative research results for mathematical topic knowledge and problem-solving skills (Ufer et al., 2008; Chinnappan et al., 2012), for methodological knowledge (Ufer et al., 2009) in the context of secondary school geometry, as well as for mathematical strategic knowledge by first studies in undergraduate contexts (Sommerhoff et al., submitted).

Corresponding research thus underlines the status of mathematical argumentation and proof skills as a resource-based cognitive skill. However, it is currently unclear what this implies for educational strategies to support mathematical argumentation and proof skills and its resources. In particular, prior research has underlined that training mathematical argumentation and proofs skills directly by working on proof (construction) tasks is not particularly effective (e.g., Weber, 2003; Selden and Selden, 2008, 2012). This result is often attributed to the lack of required resources (see Selden and Selden, 2008). Moreover, it appears possible but rather intricate to acquire the lacking resources while working on proof tasks without explicitly addressing them—solving respective tasks is already demanding for students. It thus appears more likely that approaches explicitly focusing and supporting the different resources as well as their application in the context of proof tasks may be an effective way of supporting students’ resources as well as their mathematical argumentation and proof skills.

## The Current Study

As pointed out in *Theoretical Background*, mathematical argumentation and proof skills represent a resource-based skill that has multiple underlying skills whose availability is important. Our study is a first step to explore how acknowledging the resources underlying a resource-based cognitive skill can be functional in supporting the learning of the underlying resources *as well as* the resource-based cognitive skill itself. For this, we take up the part-/whole-task debate from instructional design (Anderson et al., 1996; Lim et al., 2009; Branch and Merrill, 2011) in the pursuit of evidence for the feasibility and respective benefits of a *sequential* (analog to the part-task approach) and *concurrent* (analog to the whole-task approach) approach for supporting students’ resource-based cognitive skill and its underlying resources.

This is done by examining students’ mathematical argumentation and proof skills, which comprise a resource-based cognitive skill with mathematical topic knowledge (MTK), methodological knowledge (MK), mathematical strategic knowledge (MSK), and problem-solving skills (PSS) as underlying resources as suggested by prior research. In a quasi-experimental study with university mathematics students, we investigated whether supporting each of the four resources sequentially one after the other or supporting the resources concurrently in the context of mathematical proofs yields (higher) learning gains on the resources as well as on overall mathematical argumentation and proof skills.

The research questions driving the study are the following:

RQ1 **What are the effects of a sequential vs. a concurrent instructional approach on the *resources* of mathematical argumentation and proof skills?**
*Hypothesis:* We expected positive effects on the resources for both approaches. Moreover, we expected that the sequential approach is superior to the concurrent approach in supporting the resources of a resource-based cognitive skill.
*Argument:* Each of the resources for mathematical argumentation and proof skills as well as their utilization within argumentation and proof processes are already quite complex. Shortcomings of students regarding prior knowledge, problem-solving skills, and other aspects have repeatedly been reported (e.g., Harel and Sowder, 1998; Selden, 2011; OECD, 2014). Based on this high complexity of the “parts,” the results by Naylor and Briggs (1963) imply that a sequential approach should be better suited to support these resources. This appears highly plausible, as acquiring multiple complex resources at the same time may prove overly demanding for students as they have to process too much information for too many different resources simultaneously. The idea of instructional clarity supports this, as the sequential condition covers each resource individually and thus should lead to a higher instructional clarity, which in turn should be beneficial for the improvement of students’ resources.
RQ2 **What are the effects of a sequential vs. a concurrent instructional approach on *overall mathematical argumentation and proof skills*?**
*Hypothesis:* We expected the concurrent approach to yield higher or at least comparable learning gains compared to the sequential approach.
*Argument:* The hypothesis is implied by the results from Naylor and Briggs (1963), as overall mathematical argumentation and proof skills require a high degree of task or rather “resource organization,” that is, the underlying resources need to be purposefully combined and applied when working on mathematical proof tasks. Accordingly, an approach integrating the resources and thereby allowing students to directly experience the concurrent coordination and application of the resources within mathematical proof tasks should be favorable and lead to integrated learning. This is further supported by a prior review on part-task practice (Wickens et al., 2013) that revealed smaller effects of part-task training when parts have to be used concurrently, which holds for the resources underlying mathematical argumentation and proof skills.
Furthermore, the sequential approach requires students to later, that is, after learning about each resource, integrate the various resources and apply them purposefully when constructing mathematical proofs. As this does not arise as naturally as in the concurrent approach, where the resources are already used in an integrated way during training, this could pose another obstacle for students following a sequential approach and may actually hinder learning overall mathematical argumentation and proof skills. In line with this argumentation, situated learning theories (Brown et al., 1989; Lave and Wenger, 1991) also suggest that students should rather benefit from the authentic, meaningful combination of resources as opposed to addressing them individually.

Beyond these research questions quantitatively comparing both approaches, we were interested in *how* the expected learning gains on the resources could shape participants’ proof construction attempts and lead to the observed results for the research questions. Here, we were primarily interested in qualitative insights as to how the resources can be used and integrated by students in the concurrent condition and if this integration could lead to productive synergistic effects.

## Method^1^

### Design, Participants, and Context

We adopted a quasi-experimental pre–post design with two conditions, corresponding to the sequential and concurrent approach. The intervention was offered as a voluntary course for mathematics university students from one of the largest German universities and was entitled “Mathematical proofs: That’s how to do it!,” which was aimed toward undergraduate students after their first semester. A total of 45 students (18 male, 27 female, *m*_age_ = 20.82) participated in the study. Of these, 36 were first year and 9 were second year students who were either enrolled in a mathematics bachelor’s program or a teaching degree for secondary education. One can assume that all participants had participated in proof-based real analysis lectures, giving the students the necessary foundation for the course. In contrast to mostly calculation-based ‘calculus’ courses that include only some proofs, these lectures are purely proof based and focus on the creation of an axiomatic, deductively derived theory. However, the courses are not explicitly designed to be ‘introduction to proof’ courses, but mathematical proofs are mostly introduced in a ‘learning by observing/doing’ manner, mostly without explicitly covering or even disentangling different aspects of proofs or different resources needed for proofs. A typical book reflecting the lectures is from Amann and Escher (2005).

Twenty-one students participated in the sequential condition, while 24 students participated in the concurrent condition. Participants’ final school qualification grade (*M* = 1.92^2^, *SD* = 0.52), as well as their final high-school grades in mathematics (*M* = 1.86, *SD* = 0.56), were in between the best and second-best grade.

### Procedure

The course was scheduled across three consecutive days and consisted of four 2-h intervention sessions plus two sessions for pretest and posttest (i.e., two sessions per day). Without being aware of the difference, participants could choose to participate in one of both parallel groups, each representing one of the instructional conditions. The course was conducted by two experienced instructors with a mathematics and mathematics education background. Instructors swapped groups in the middle of the intervention to counter instructor effects.

The content of the course was based on topics and proofs from *proof-based real analysis*, an introductory topic in undergraduate mathematics. Both conditions covered the same teacher input, content, tasks, and time on task. Yet, tasks and content were arranged in a different order according to both conditions.

**FIGURE 3:**
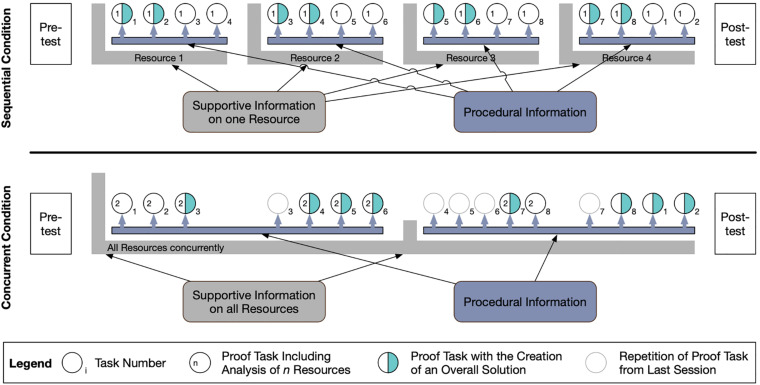
Instructional design used for both conditions within the intervention.

To teach the individual resources in both conditions, we adopted a 4C/ID-inspired instructional design (van Merriënboer and Kirschner, 2007; van Merriënboer, 2013). Following this design, the teaching of the resources consisted of an initial input phase with information on the resources, giving both a theoretical background as well as information on why, how, and when they are important during activities related to mathematical argumentation and proof. This was combined with a short list of elaboration and monitoring prompts that were distributed to the students (e.g., MTK: “Excerpt all important objects and properties from the task, explain these in your own words, and compare them to the formal definition.;” MSK: “Search the task for keywords that you know from other tasks. What methods did you use there?”). The prompts represented procedural information that students could use while solving proof construction tasks. They were intended to scaffold the use and application of the individual resources during these tasks, to enhance students’ analysis of the tasks according to each resource, and to stimulate students to elaborate and reflect on each resource. To show how these prompts can be purposefully applied, the instructor demonstrated and trained their use with the students based on an example task (for each resource individually on one task in the sequential condition; simultaneously in each of the four tasks in the concurrent condition). After these input and training phases, which lasted about 15 min per resource (distributed over four sessions in the sequential condition; clustered in two parts in the concurrent condition; see Figure 3), students worked on proof construction tasks individually trying to implement what they had just learned and find more effective approaches to proof construction than they had before.

The 4C/ID-inspired instructional design was used for two reasons: First, each of the resources is characterized by a lower *task organization*, that is, the aspects within the resources require less organization as compared to mathematical argumentation and proof tasks and therefore should benefit from a rather comprehensive instructional approach (Naylor and Briggs, 1963). Second, we parallelized instruction on the individual resources for both conditions, as the research questions relate to effects of their sequential or concurrent teaching, that is, the arrangement of the resources within the course, and differences regarding the teaching of the individual resources between both conditions may have biased results.

#### The Sequential Condition

The sequential condition was intended to support each of the four different resources separately. Accordingly, the course was split into four sessions of 2 h each for this approach, which each focused only one of the four resources (Figure 3, upper section).

After the input phase, students worked on exactly four tasks in each session and analyzed them, focusing on the one resource that was covered during that session. Each task was then picked up in a second session and analyzed regarding the resource covered during that session. Additionally, students solved the task itself and created an overall solution of the task (including the correct solution of the task as well as the analyses regarding each of the two resources), which was discussed with the instructor.

During students’ analyses and their work on the tasks, the instructor gave guidance, provided procedural information, and gave students hints to use specific prompts from the provided list.

#### The Concurrent Condition

The concurrent condition also consisted of four 2-h sessions to have similar learning times/time on task for both conditions. However, each session included all four resources, providing students with the opportunity to integrate the individual resources and see connections among them.

In this condition, the content of the four input phases in the sequential condition were rearranged to two input phases at the beginning of the first and third session (Figure 3, lower section). As all resources were covered during each session, it was necessary to give a basic amount of supportive information on all resources in the first session, so that students could work purposefully with all four resources. The remaining information was then introduced at the beginning of the third session.

Throughout the course, students from this condition worked on the same eight proof tasks used in the sequential condition, yet always analyzed them regarding two of the resources concurrently within one session. The tasks were distributed over the sessions so each resource would be covered in every session and each combination of two resources (e.g., MTK and PSS, MTK and MK, or MSK and PSS) would occur equally often. The tasks that had already been analyzed and solved (e.g., Task 3 in Session 1) were reconsidered briefly in the next session as repetitions so that each student worked on each task twice as in the sequential condition to ensure similar coverage.

The students from the concurrent condition received the same amount and kind of guidance as the students in the sequential condition.

### Instruments

Pretest and posttest of the study included scales for each of the four resources, one for students’ mathematical argumentation and proof skills, as well as for covariates and demographic data. The employed scales were adapted to the content, translated from English, or self-created if no suitable published scales were available in the literature. Except for the covariates, which were only assessed in the pretest, we used non-identic, parallelized tasks for the pre- and posttest to avoid repetition effects. We chose this approach over using identical tasks, as it was especially important for the items within the *problem solving* and the *mathematical argumentation and proof* scale to be unknown and therefore retain a problem character (e.g., Dörner, 1979; Schoenfeld, 1985).

The employed scales had been piloted prior to the reported study. Their reliability was 0.58 < α < 0.81, with 0.58 corresponding to the only scale below 0.6 (mathematical strategic knowledge) that had been assessed using only four items. As a newly developed scale for a construct that has not been assessed quantitatively before, we decided to retain the scale despite of the low reliability. This decision was backed up by better reliabilities in the pre- and posttest of the reported study (see below).

The scales contained open as well as closed items. Closed items were evaluated using mark-recognition software with a subsequent manual control. Open items were coded by two raters following theory-based coding schemes. Double coding of over 15% of the data led to an interrater reliability of κ > 0.78 (*M* = 0.93; *SD* = 0.10). For each scale, sum scores were calculated and scaled to values between 0 (worst) and 1 (best).

#### Dependent Variables

*Mathematical Topic Knowledge*. The scale was adapted from existing tests in the context of university mathematics (Wagner, 2011; Rach and Ufer, 2020) and slightly modified to fit the content area of the study. It contained eight items focusing on conceptual topic knowledge, assessing fundamental knowledge such as definitions, theorems, and properties of objects as well as their connections. It further contained five items focusing on procedural topic knowledge, assessing routine procedures as solving equations or using the formula for the geometric sum, which were required in the employed proofs throughout the course and the corresponding scale.

*Methodological Knowledge*. The scale for students’ methodological knowledge was taken from a parallel research project on the conception of proof (Sommerhoff and Ufer, 2019) and was initially based on existing scales from secondary school contexts (Healy and Hoyles, 2000; Heinze and Reiss, 2003; Ufer et al., 2009). It contained four purported proofs that included different possible shortcomings related to the nature and concept of proof (e.g., circular reasoning, unwarranted implications). Students were required to judge the validity of the purported proofs and justify their judgments.

*Mathematical Strategic Knowledge*. Mathematical strategic knowledge has, to our knowledge, not been quantitatively measured up to now. Building on the definition of the construct, we chose four typical tasks, the real analysis as the foundation for four items. The tasks were presented to students alongside four excerpts of the same task description. In a multiple-choice format, students were asked to select those excerpts that indicate a certain concept or method that would be purposeful to solve the task. In a subsequent open question, students were asked to explain their choice and describe what the excerpts would imply. Closed and open items for each task description were combined using a dichotomous consistency rating, evaluating whether the selected excerpts combined with the given explanation matched the given task.

*Problem-Solving Skills*. Students’ problem-solving skills were measured using four open items, asking students to solve problems that did not require domain-specific knowledge (neither mathematical nor from another domain), except for everyday knowledge and basic arithmetic skills. The items were then scored on a scale from 0 to 4, evaluating if the main steps for solving the problem were given and justified adequately. As heuristics are an important resource for problem solving, students’ knowledge about and their use of problem-solving heuristics was additionally assessed. Hence, students were asked how often they made use of 12 different, prototypical problem-solving strategies (e.g., means-end analysis, creating a sketch) taken from the literature (Polya, 1945) during proof construction. Each of the strategies was reflected in four Likert-scale items. Data from both aspects were combined and rescaled to 0 (minimum) to 1 (maximum).

*Mathematical Argumentation and Proof Skills*. Besides the resources, a scale for assessing students’ mathematical argumentation and proof skills consisting of four proof construction items (four tasks in the pretest, four parallelized tasks in the posttest; see Supplementary Material) was included. The tasks were chosen to be novel to the students yet reflecting prototypical tasks from real analysis lectures as well as those used within the intervention itself. The items were scored on a scale from 0 to 4, evaluating if the main ideas or steps needed for a valid proof were given and adequately justified. 0 was assigned for purported proofs that did not include a single main idea, 1 was given when at least one of the main ideas was presented, whereas the codes 2 and 3 were given if the majority and if all main ideas were present, while 4 was only given for proofs including all main ideas as well as a clear overall structure and reasoning. The scoring was (i) based on a theoretical analysis of possible solutions and important steps within these solutions and (ii) explicitly adapted to the norms established within participants’ mathematics lectures, thus reflecting the mathematical norms of early undergraduate mathematics rather than our norms as researchers.

#### Further Variables

Besides the scales for the dependent variables (resources and mathematical argumentation and proof skills), we also included a shortened scale for *conditional reasoning skills* from the literature (Inglis and Simpson, 2008) with 16 items. As conditional reasoning skills are considered fundamental for any kind of reasoning activity, important for scholarly activities across disciplines, and were also shown to significantly predict certain aspects of mathematical argumentation and proof skills (Leighton and Sternberg, 2004; Alcock et al., 2014), they were included to be used as a covariate in the later comparisons between conditions.

Finally, we gathered demographic data including gender, degree program, final school qualification grade, and final high-school mathematics grade.

### Implementation Check and Process Data

**FIGURE 4:**
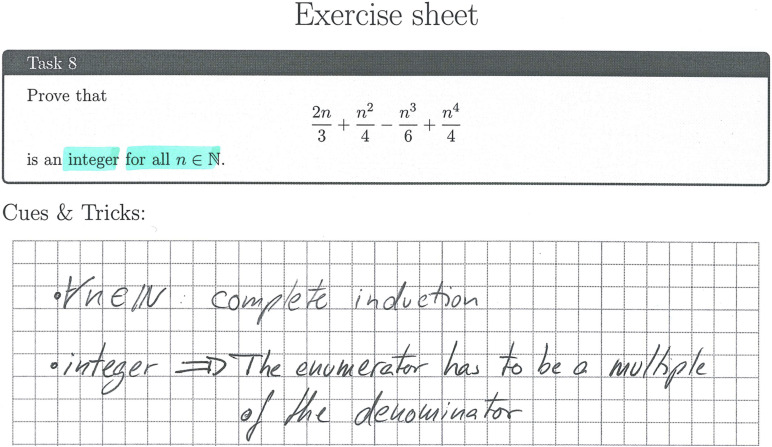
Excerpt of a student’s exercise sheet showing an analysis regarding mathematical strategic knowledge (translated).

To check the implementation within both conditions and to survey process data, students received prefabricated exercise sheets to work with for all tasks and analyses regarding the resources. The sheets were gathered and digitalized after every session throughout the intervention (see Figure 4 for an excerpt of an exercise sheet showing the analysis of a task regarding mathematical strategic knowledge). Subsequently, it was checked whether students had explicitly analyzed the task regarding the resources and whether the analysis was done on a meaningful or a superficial level (dichotomous rating).

Additionally, a reflection scale on the content covered by the course was created for the posttest, probing students about several topics that may or may not have been covered by the course (e.g., “I think I learned a lot regarding problem solving”). To check that both conditions did indeed convey an individual respectively concurrent conception of the resources, students were also asked how separated they perceived the different resources during the intervention (“I think the course separated the individual prerequisites of proving well.”).

### Statistical Analysis

Analyses of covariance (ANCOVAs) were calculated for each resource including conditional reasoning skills and the pretest results of the respective resource as covariates to examine the effectiveness of both approaches regarding the four resources. We refrained from calculating an overall multivariate ANCOVA (MANCOVA) as both, not including the pretest scores on the resources as well as “throwing in” all pretest scores as covariates seemed inappropriate, both theoretically and from a methodological point of view.

The second research question was examined similarly by using an ANCOVA with students’ mathematical argumentation and proof skills as dependent variable and conditional reasoning skills and the corresponding pretest results as covariates. To further analyze the possible influence of prior attainment on the learning gains, a median split based on the pretest results on mathematical argumentation and proof skills was calculated, and the effects of each condition on each subgroup were examined.

Additionally, we calculated Hedges’ *g*_*av*_ as a measure for longitudinal effect sizes (Lakens, 2013) to estimate the effectiveness of either approach on the resources and on mathematical argumentation and proof skills beyond their mere significance, as the number of participants in each group was low, especially regarding the median split.

For the supplementary qualitative analysis on how the concurrent approach can shape participants’ proof construction attempts, a prototypical (based on the pretest scores) participant of the concurrent condition was randomly selected. Her proof construction attempts from Task 7 (see Figure 6), which was covered in the second last and last intervention sessions, were then qualitatively analyzed to showcase the possible effects of the concurrent approach, however, not implying any generality of these exemplary findings. The qualitative analysis should thus be understood as an existence proof on how the different resources can be synergistically integrated in the concurrent condition.

## Results

### Implementation Check

An analysis of the documents used throughout the intervention confirmed that students in both conditions actively analyzed the tasks regarding the respective resources and used the provided prompts to elaborate and reflect on the resources. Overall, 92.5% of the suggested analyses regarding the resources were completed, 1.9% were missing, and 5.6% were done on a superficial level.

This indication of a correct implementation was further supported by the results of the posttest: A related samples Friedman two-way analysis of variance by ranks on the reflection scale, which probed students about several topics that may or may not have been covered by the course, showed overall significant differences between students’ answers on the covered topics [χ^2^(6) = 89.048, *p* < 0.001). *Post hoc* Dunn–Bonferroni tests showed significantly lower values for both topics not covered during the course (“beliefs,” “quantifier logic”) in comparison to those covered by the course.

Furthermore, a Mann–Whitney *U* test on students’ rating of the perceived separateness of the resources showed the expected significant difference (*U* = 327.0, *p* = 0.029; *M*_*sequential*_ = 3.0 and *M*_*concurrent*_ = 3.3), indicating that the participants of the sequential condition perceived the resources as more separated than students from the concurrent condition.

### Descriptive Results

The employed scales in the pre- and posttest (Table 1) showed acceptable values and variances as well as no signs of floor or ceiling effects. Cronbach’s alpha was acceptable 0.64 < α < 0.84 for all scales in pre- and posttest, in particular showing better values for mathematical strategic knowledge (pretest: 0.64; posttest: 0.71). No indications for violations against normal distribution or equality of variances were found for resources and mathematical argumentation and proof skills.

**TABLE 1:** Mean values for the scales obtained for both conditions in pre- and posttest.

	Sequential				Concurrent			
	Pretest		Posttest		Pretest		Posttest	
	*M*	*SD*	*M*	*SD*	*M*	*SD*	*M*	*SD*
Mathematical Topic Knowledge	0.33	0.17	0.45	0.21	0.40	0.16	0.49	0.14
Methodological Knowledge	0.40	0.16	0.54	0.14	0.49	0.17	0.55	0.16
Mathematical Strategic Knowledge	0.35	0.16	0.57	0.16	0.39	0.17	0.69	0.18
Problem-solving Skills	0.53	0.10	0.53	0.09	0.54	0.09	0.57	0.10
Mathematical Argumentation and Proof Skills	0.34	0.14	0.29	0.14	0.36	0.18	0.32	0.14

*All scales ranged from 0 (minimum) to 1 (maximum).*

Pearson correlations for each pair of parallelized scales [MTK, MK, MSK, PS, MA&P] were calculated to safeguard against possible problems regarding the comparability of the parallelized pre- and posttest scales. These showed moderate to strong, highly significant correlations [*r*(43) = 0.48 − 0.69, *p* ≤ 0.001].

The results of the pretest regarding the dependent variables, that is, the resources as well as students’ mathematical argumentation and proof skills, suggested that both conditions were comparable prior to the intervention (Table 1). This was confirmed by calculating independent samples *t*-tests comparing both conditions for each of the resources and mathematical argumentation and proof skills. None of the tests gained significance [*t*(43) < 1.60, *p* > 0.118], solely methodological knowledge slightly approached significance [*t*(43) = 1.75, *p* = 0.088] in favor of the participants in the concurrent condition.

The same insignificant differences were found for students’ conditional reasoning skills [*t*(43) = 0.36, *p* = 0.720], which were subsequently used as a covariate, as well as for the demographic data gathered.

### Effects on the Resources (RQ1)

The descriptive results of the posttest (Table 1) showed learning gains within both conditions for most resources, leading to pre–posttest effect sizes of *g*_*av*_ = 0.35 − 1.73 (Table 2). Solely students’ problem solving showed small to no gains depending on the experimental condition (*g*_sequential,av_ = 0.00 and *g*_concurrent__,av_ = 0.25).

**TABLE 2:** Longitudinal effect sizes (Hedges’ g_av_) for both conditions.

	MTK	MK	MSK	PSS	MA&P
Sequential	0.64	0.90	1.36	0.00	–0.30
Concurrent	0.58	0.35	1.73	0.25	–0.27

**FIGURE 5:**
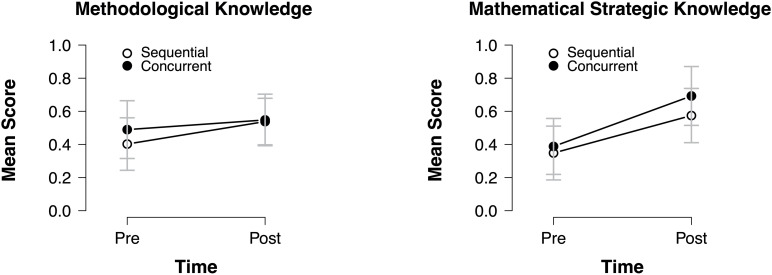
Effects of both approaches on methodological knowledge (left) and mathematical strategic knowledge (right).

Comparing the descriptive results of the posttest between both conditions (Table 1), slightly higher mean scores for all resources within the concurrent condition could be observed, which could be an indication for higher learning gains in this condition. To statistically control these descriptive findings, univariate ANCOVAs on the posttest results of each resource were calculated while controlling for conditional reasoning skills *and* the respective pretest score. Results revealed a significant difference on mathematical strategic knowledge [*F*(1,41) = 5.19, *p* = 0.028, η^2^ = 0.112], confirming significantly higher learning gains in the concurrent condition. All other ANCOVAs were insignificant [*F*(1,41) < 1.538, *p* > 0.222], thus not confirming the descriptive differences between both conditions. The significant result of the ANCOVA for mathematical strategic knowledge was also reflected in the (significant) longitudinal learning gains [paired samples *t*-tests: sequential: *t*(20) = −10.19, *p* < 0.001; concurrent: *t*(23) = −7.48, *p* < 0.001]. Although the concurrent condition showed larger effects (*g*_sequential__,av_ = 1.36 and *g*_concurrent__,av_ = 1.73; Figure 5, left side), adding an interaction in the ANCOVA turned out insignificant [*F*(1,40) = 2.56, *p* = 0.118, η^2^ = 0.060], thus not confirming the descriptive differences in pre–post effect sizes.

Although no statistically significant effect in the ANCOVA for methodological knowledge was found, descriptive data and effect sizes gave a first indication for a between-conditions effect (Figure 5, right side): The gains in the sequential condition [*g*_sequential__,av_ = 0.90; *t*(20) = −4.238, *p* < 0.001] appear to be larger than in the concurrent condition [*g*_concurrent__,av_ = 0.35; *t*(23) = −1.66, *p* = 0.110], indicating that students in the sequential condition caught up with the students from the concurrent condition. Adding an interaction in the ANCOVA again turned out insignificant [*F(*1,40) = 1.21, *p* = 0.278, η^2^ = 0.030].

### Effect on Students’ Argumentation and Proof Skills (RQ2)

The descriptive results of the pretest and posttest for students’ mathematical argumentation and proof skills (see Table 1) and the corresponding longitudinal effect sizes in both conditions (*g*_sequential__,av_ = −0.30 and *g*_concurrent__,av_ = −0.27) showed slightly lower scores. Descriptive data thus suggests that the tasks in the posttest were more difficult for students, although they had been designed to be parallel in structure and comparable in difficulty to the pretest (see also *Discussion*; see Supplementary Material for the items). A one-way ANCOVA on students’ mathematical argumentation and proof skills in the posttest, controlling for students’ conditional reasoning skills and their pretest results on mathematical argumentation and proof skills, showed no significant difference between both conditions [*F*(1,41) = 0.144, *p* = 0.706].

To examine the longitudinal effects on mathematical argumentation and proof skills in more detail, we performed an exploratory analysis comparing students with different prior attainment, as prior research suggested its possible role for the effectiveness of either condition (Salden et al., 2006; Lim et al., 2009). For this purpose, two groups were formed using a median split according to students’ pretest results on mathematical argumentation and proof skills. The split resulted in four groups, a weaker and a stronger group for both instructional approaches. Calculating the longitudinal effects for the four groups showed mixed effects of the intervention (Table 3).

**TABLE 3:** Longitudinal effect sizes (Hedges’ *g*_av_) on students’ mathematical argumentation and proof skills for the median-split groups.

		Number of students	Pretest		Posttest		Effect size *g*_av_
			*M*	*SD*	*M*	*SD*	
Weaker	Sequential	11	0.22	0.09	0.22	0.10	–0.06
	Concurrent	8	0.15	0.11	0.23	0.10	0.71
Stronger	Sequential	10	0.46	0.06	0.38	0.14	–0.84
	Concurrent	16	0.47	0.08	0.36	0.14	–0.95

Data suggest that students’ prior attainment had an impact on the effectiveness of the instructional approaches and may indicate an expertise reversal effect: Initially, stronger students actually showed a negative development regarding their mathematical argumentation and proof skills from pre- to posttest, whereas initially weaker students outperformed them. Although group sizes are small, the initially weaker students from the concurrent condition show a quite positive development (*g*_av_ = 0.71) with a medium to large positive effect, whereas the initially weaker students’ mathematical argumentation and proof skills did not change profoundly in the sequential condition (*g*_*av*_ = −0.06). In contrast, differences between both conditions for the initially stronger students appear to be much smaller.

### The Concurrent Condition—An Illustration of Effects

The exploratory analysis based on the median split revealed first signs of an expertise reversal effect regarding students’ mathematical argumentation and proof skills (not for the resources), that is, initially stronger students benefit less and even show a negative development based on the resource-based interventions as compared to initially weaker students. Especially weaker students in the concurrent condition seemed to benefit from the intervention, as the concurrent focus on multiple resources may have led to a better integration and handling of the resources in argumentation and proof tasks. Even though data does not allow a further statistical underpinning of this claim, a qualitative examination may provide insights into the possible effects of the concurrent approach for students with lower initial argumentation and proof skills. For this purpose, we provide a deeper analysis of a proof construction attempt by Leia (ficticious name), a prototypical student (based on her pretest scores) from the “weaker–concurrent” group, which she had created during the second last session of the intervention. Leia was 23 years old, in the first year of her bachelor mathematics studies. She failed both exams from the first semester, which drew heavily on proof construction.

**FIGURE 6:**
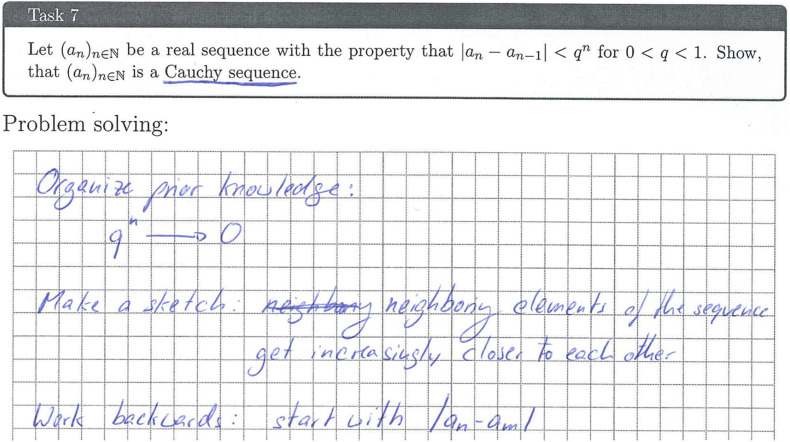
Task description and Leia’s notes regarding problem solving (translated).

**FIGURE 7:**
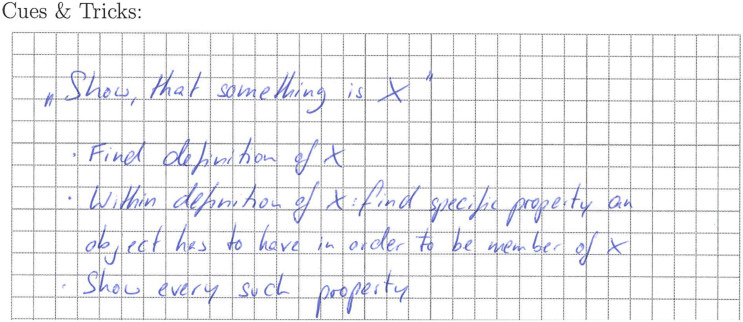
Leia’s notes regarding mathematical strategic knowledge (translated).

Leia’s work on the analysis of the given proof task regarding the resource *problem solving* (Figure 6) shows three main thoughts, each fitting to one of the elaboration and monitoring prompts given to the students. The first two mirror her attempts to make sense of the meaning of the property of the given sequence, which seems to work out to a certain degree as the second point correctly reflects the given property. The third point shows that she has created a plan for solving the task, even before actively trying to do so in her actual proof attempt. That is, she plans to use the general *problem-solving heuristic* of working backwards, here starting from the defining property of a Cauchy sequence (given in mathematical notation). This strategy matches her work regarding *mathematical strategic knowledge* (Figure 7; called “cues and tricks” in the intervention), which focuses on the analysis of the task formulation and its consequences for the solution of the task. By concentrating on the structural parts of the given task, Leia unveils its type, referring to it as a “Show, that something is X” task. She then lays out a broad idea on how to solve this type of task by finding the properties that have to hold for an object to be a member of class “X” and then showing that these properties hold. Her work regarding this resource only represents a small aspect of mathematical strategic knowledge and is very procedural (regarding the solution of the task). However, it mirrors the heuristic of working backwards mentioned in her problem-solving analysis from a mathematical strategic perspective, thus aligning domain-specific and domain-general strategies.

**FIGURE 8:**
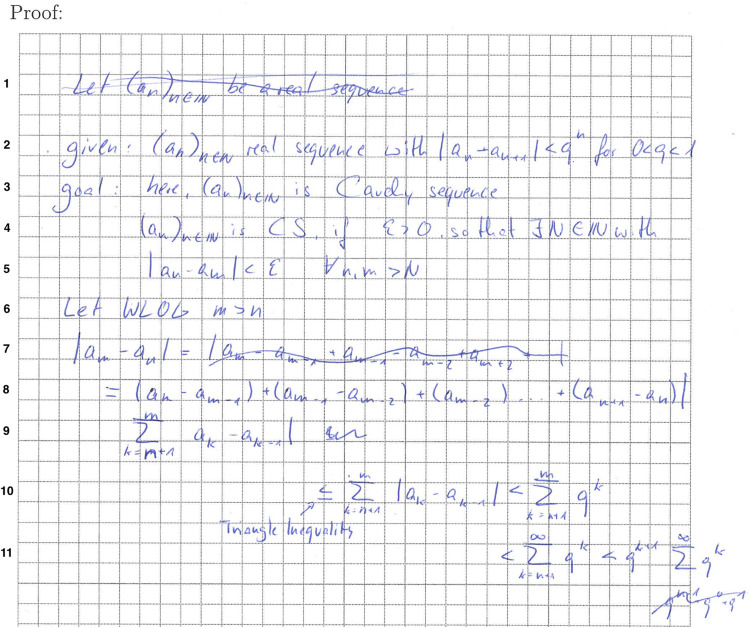
Leia’s proof attempt (translated; line numbers added).

After carrying out both analyses, Leia starts her proof attempt (Figure 8). Apparently, she jumps quickly into the proof but is unsatisfied by her first approach and crosses it out (Line 1). As the crossed-out line is correct and resembles a reasonable approach for the task, it may be assumed that Leia hesitates because she wants to stick to the information and procedures given to her in the intervention, asking her to clarify what is given and, in particular, her goal. Leia’s behavior may thus be interpreted as a hesitation or conflict to pursue her prior approaches to proof construction, which may be even more profound and difficult for students with higher prior attainment, who are more convinced of their prior approaches.

Starting in her third line, she then lays out the definition of a Cauchy sequence (with one minor error in Line 4), which she then uses in her actual proof attempt, starting from Line 6. Here, she can successfully reduce the property of a Cauchy sequence to the property of the given sequence (Lines 8–10) but fails to explicate the last proof step and conclude that the resulting term converges to zero as *n* increases.

Leia’s work exemplifies that especially low-attaining students may have benefitted from a structured approach to mathematical argumentation and proof tasks. In her case, the explicit discussion of aspects of the task related to the resources required for the task appear to have helped her to plan her problem-solving process and to purposefully integrate and apply her mathematical topic knowledge about Cauchy sequences in her planning process. This may be seen as a result of the concurrent focus on two resources, problem-solving skills and mathematical strategic knowledge, as the conjunction of the results regarding both resources appear to have shaped her solution.

Leia’s work thus highlights that the intervention had a learning effect and that she implemented her new knowledge on how to approach mathematical proof construction tasks. Still, her work also highlights that this newly acquired, resource-based approach can also constrain solution processes to a certain degree. She did not pursue her first approach to proving the statement (Line 1) but seems to have changed her approach. Apparently, the newly acquired knowledge was not sufficient for her to adequately judge how productive her attempt in Line 1 was. This may point to an insufficient integration of the new knowledge and skills and that using them in specific proof construction tasks was still challenging enough to prevent complete and efficient success, something that may be expected after such a short intervention and may eventually disappear with more practice and routine.

## Discussion

Our intervention study examined two instructional approaches to support the learning of mathematical argumentation and proof skills as a resource-based skill while also aiming at learning benefits for the included underlying resources themselves. For this, a *sequential* approach focusing on each resource individually one after the other and a *concurrent* approach focusing on the resources concurrently, both which were inspired by the part-/whole-task debate from instructional design (see e.g., Lim et al., 2009), were compared. Due to the low sample size, especially in the median split groups, the study’s results have to be handled with care and can only be interpreted as first evidence regarding the effectiveness of resource-based instructional approaches. However, power analysis confirms that the ANCOVAs employed in this study to compare effects between conditions should have been suited to identify large effects (*f* > 0.43) with more than 80% power. Moreover, results from this study—even if some are only tentative—will be essential for further research, as multiple effects and possible mechanisms have been highlighted, which can now be addressed more specifically by future research.

### Effects on the Resources

The analyses of the results revealed that explicit training of the included resources of mathematical argumentation and proof skills lead to notable learning gains regarding some of the resources, while others only showed a slightly positive development. The longitudinal effect sizes indicate especially high positive effects for mathematical strategic knowledge. These may reflect that mathematical strategic knowledge was not explicitly covered during the participants’ university instruction on mathematics so that initial learning gains are easy to achieve. They may, however, also be an indication that mathematical strategic knowledge is indeed an important, so far under researched resource of mathematical argumentation and proof skills.

Comparing the impact of both approaches on the four resources, no overall significant differences for students’ resources could be found. Solely students’ mathematical strategic knowledge showed a significant difference in favor for the concurrent condition. Although our assumption was that the sequential approach would be superior for the learning of the resources, this result appears reasonable: Mathematical strategic knowledge refers to knowledge about cues within mathematical tasks that lead to promising methods or concepts to tackle the tasks and further refers to knowledge about strategies to solve these tasks (Weber, 2001). It therefore is related to creating the problem space, identifying operators therein, and choosing an operator that may be useful to accomplish the task (see Newell and Simon, 1972). The successful use of mathematical strategic knowledge therefore corresponds to a rather comprehensive view of tasks and is not only limited to certain aspects of the task. In particular, mathematical strategic knowledge shows multiple connections to the other resources, as for example, mathematical topic knowledge is needed to create the problem space and identify the operators. Further, methodological knowledge is needed to identify what a goal state for the problem is supposed to entail. Accordingly, the concurrent approach may be especially beneficial for mathematical strategic knowledge as implied by the data, as it may emphasize and strengthen relations to other resources.

### Effects on Mathematical Argumentation and Proof Skills

Results on mathematical argumentation and proof skills are quite surprising, as longitudinal effect sizes suggest a slightly negative (yet not significant) development based on the intervention. Multiple possible explanations for this effect arise, each of which will have to be addressed by future research: The effect may be a methodological artifact of a more difficult posttest. It may however, also reflect that mathematical argumentation and proof skills are highly complex (especially compared to those skills usually addressed in the part-/whole-task debate) and that the relatively short intervention may not have sufficed to transfer the observed learning gains on the resources to mathematical argumentation and proof skills. Finally, the observed expertise reversal effect (e.g., Kalyuga et al., 2003) may be responsible for this overall development as the approach is simply better suited for even weaker students.

Despite the inconclusive overall development, data are still suitable to compare the effects of both approaches on mathematical argumentation and proof skills as intended by the study. Here, an ANCOVA comparing the posttest results did not show a significant difference between both approaches regarding students’ mathematical argumentation and proof skills. Still, examining this result more closely by forming groups of differing prior attainment revealed interesting effects: Compared to initially stronger students, weaker students showed a better development regarding their mathematical argumentation and proof skills. Here, especially the students from the concurrent approach could benefit, suggesting that at least for initially weaker students, the integration of the individual resources and their concurrent application within mathematical proof tasks is important to support overall mathematical argumentation and proof skills. This is also exemplified in the qualitative analysis of Leia, an initially weaker student from the concurrent approach. Her work on the resources and the overall task suggests that working concurrently on both resources was beneficial for her to derive a solution for the task and that she was able benefit from the structured approach to the task by using the resources.

## Conclusion and Outlook

The current study highlights that acknowledging the resource-based nature of a cognitive skill can inspire instruction and raises new questions for mathematics research and education. Our study creates first knowledge on the effectiveness of two *resource-based instructional approaches*, both of which explicitly acknowledge the resources underlying a cognitive skill, in the context of mathematical argumentation and proof skills.

Results suggest that, in the case of mathematical argumentation and proof skills, the sequential and the concurrent approach can both be used to support students, at least regarding the resources. Here, the approaches yielded mostly similar learning gains, both regarding the substantial short-term learning gains for mathematical strategic knowledge as well as regarding the positive, yet less pronounced, effects for the other resources. Regarding mathematical argumentation and proof skills, results of the short intervention do not show the expected learning gains, and both approaches did not show large differences as implied by the part-/whole-task debate (van Merriënboer and Kester, 2007; Branch and Merrill, 2011) but are mostly comparable in learning gains. In particular, the concurrent work on the resources appears to not have led to the expected superior integration of the resources and their better application within mathematical proof tasks in comparison to the sequential condition. This may be due to participants’ struggles to implement the new approach and focus explicitly on the resources while solving the tasks so shortly after the intervention. This is also highlighted by the qualitative example of Leia: Even for those successful in implementing the approach, there appear to be certain struggles when starting to solve the task and shifting from former proof-construction approaches to rather resource-based approaches.

However, contrary to these short-term findings, long-term learning effects may be more positive when students have been better trained and internalized the approaches. Although this hypothesis will have to be confirmed by future research, it is supported by somewhat similar research from educational psychology (Rittle-Johnson and Star, 2011; Ziegler and Stern, 2014) focusing on sequenced learning (similar to the sequential condition) and contrasting learning (similar to the concurrent condition). Results reveal that the contrasting condition showed equal short-term learning as the sequenced condition but improved long-term learning. It thus appears plausible that the concurrent approach may be more effective regarding long-term learning.

Overall, further studies exploring the effectiveness of resource-based instructional approaches are needed: (i) quantitative studies with larger samples to obtain higher statistical power, (ii) qualitative studies focusing on the processes during the intervention as well as students’ proof construction processes after the intervention in order to identify how learning gains on the resources can be transferred to overall mathematical argumentation and proof skills, and (iii) long-term studies examining the observed differences regarding prior attainment and the benefits for weaker students in the long run. Finally, further studies should put even more focus on mathematical strategic knowledge, which showed high learning gains in this study but has the weak point that it was quantitatively operationalized for the first time in this study.

Another reason for further research and a possible limitation of this study is the selection and operationalization of the resources included in the reported study. As pointed out in the theoretical background and method section of this paper, there is reasonable evidence to assume that the four included resources actually are resources of mathematical argumentation and proof skills and explain the majority of variance in students’ mathematical argumentation and proof skills. However, other resources, for example, beliefs (e.g., Schoenfeld, 1985), could also have been investigated, and also other operationalizations of the resources could have been used. Future studies focusing on different sets of resources and different operationalizations could strengthen the results, both regarding the effectiveness of the instructional approaches as well as regarding the status of the resources.

Finally, including a control condition would be desirable in future studies to consolidate the results of this study, especially regarding the possibly negative development of mathematical argumentation and proof skills. However, there is no generic choice how to implement such a control group, as most resources are not explicitly taught in “regular” university mathematics courses in Germany. We would therefore rather propose to compare intervention approaches acknowledging the underlying resources with several other approaches, not explicitly taking the resources into account (e.g., Moore, 1994; Selden and Selden, 1995; Heinze et al., 2008). Outcomes could show whether acknowledging the underlying resources is beneficial for supporting students’ learning or whether other approaches show superior effects. Here, special attention should be paid to the comparability of the interventions, for example, by using academic learning time, time on task, or equivalent as a general measure.

Our studies’ main goal was to explore whether two different approaches inspired by research from instructional design (sequential and concurrent approach) could be purposefully transferred to mathematics education and the context to mathematical proofs in order to support mathematical learning. In this regard, we were interested if both approaches would yield different learning gains regarding a resource-based skill and its resources. Findings reveal that the tenet of the part-/whole-task debate (Anderson et al., 1996; Branch and Merrill, 2011), that whole-task approaches are favorable in the context of complex skills, cannot be transferred directly, at least not regarding short-term effects.

However, the study indicates that including the resources into instruction supporting mathematical argumentation and proof skills as a prototypical resource-based skill is highly valuable for the learning of the resources. Moreover, the effects for the initially weaker students in the concurrent condition underline that supporting these resources can have substantial positive effects on students’ mathematical argumentation and proof skills. In this regard, it appears as if the concurrent approach investigated in this study may be especially suitable to support students that struggle substantially with learning proof construction.

## Data Availability Statement

The raw data supporting the conclusions of this article will be made available by the authors, without undue reservation.

## Ethics Statement

Ethical review and approval was not required for the study on human participants in accordance with the local legislation and institutional requirements. The patients/participants provided their written informed consent to participate in this study.

## Author Contributions

All authors listed have made a substantial, direct and intellectual contribution to the work, and approved it for publication. All authors worked together in preparing and conceptualizing the study design. DS designed all materials, collected the data, created analysis tools, performed the analysis, and wrote the manuscript. IK counseled regarding instructional design. SU counseled regarding the measurement and conceptualization of MAP skills and the resources and reviewed the materials.

## Conflict of Interest

The authors declare that the research was conducted in the absence of any commercial or financial relationships that could be construed as a potential conflict of interest.

## Footnotes

1 Further details regarding the design of the study, the teaching materials, the employed instruments, and the obtained data can be requested for research purposes (e.g., replication/reanalysis) from the authors.

2 Grades are scaled from 1 to 4, with 1 being the best grade.
