# Supplementary material for: Supporting Mathematical Argumentation and Proof Skills: Comparing the Effectiveness of a Sequential and a Concurrent Instructional Approach to Support Resource-Based Cognitive Skills
Source: Front Psychol. 2021 Jan 21;11:572165. doi: 10.3389/fpsyg.2020.572165 (PMC7858258; doi:10.3389/fpsyg.2020.572165)
Supplement: Supplementary file 1 [file Data_Sheet_1.pdf]

Supplementary Material

1 SCALES FOR MATHEMATICAL ARGUMENTATION AND PROOF SKILLS (PRE- AND POSTTEST)

1.1 Pretest

The following four tasks were used to measure mathematical argumentation and proof skills in the pretest. The general intruction for participants (for all tasks) was "*Solve the following tasks as completely as possible and give arguments for your steps.*"
The descriptions provided for each task (e.g., "involving complete induction") were not displayed in the actual test and are included to illustrate how the tasks were parallelized.

1.1.1 Task 1 (Involving complete induction)

Show that the following equation holds for all $n \in \mathbb{N}$ with $n \geq 1$:

$$\sum_{k=1}^{n}(2k-1) = n^2$$

Justify each of your steps.

1.1.2 Task 2 (Involving partial fraction decomposition)

Determine if the following series is convergent and, if so, determine its limit. Justify each of your steps.

$$\sum_{k=1}^{\infty} \frac{1}{(k+1) \cdot (k+2)}$$

1.1.3 Task 3 (Involving a proof by contradiction)

Proof the following claim and justify each of your steps:

If the series $\sum_{k=0}^{\infty} a_k$ is convergent, the sequence $(a_k)_{k\in\mathbb{N}}$ is convergent with limit 0.

1.1.4 Task 4 (Involving the recursive definition of a sequence)

Let $(a_n)_{n\in\mathbb{N}}$ and $(b_n)_{n\in\mathbb{N}}$ be sequences of real numbers with $a_1 = 2, a_{n+1} = 2a_n$ and $b_n = 2^n$ for all $n \in \mathbb{N}$.
Show that the following holds for all $n \in \mathbb{N} : a_n = b_n$.

Justify each of your steps.

1.2 Posttest

The following four tasks were used to measure mathematical argumentation and proof skills in the posttest. The general intruction for participants (for all tasks) was ”*Solve the following tasks as completely as possible and give arguments for your steps.*”
The descriptions provided for each task (e.g., ”involving complete induction”) were not displayed in the actual test and are included to illustrate how the tasks were parallelized.

1.2.1 Task 1 (Involving complete induction)

Show that the following equation holds for all $n \in \mathbb{N}$:

$$\sum_{k=0}^{n} \frac{1}{2^k} = 2 \cdot \left(1 - \frac{1}{2^{n+1}}\right)$$

Justify each of your steps.

1.2.2 Task 2 (Involving partial fraction decomposition)

Determine if the following series is convergent and, if so, determine its limit. Justify each of your steps.

$$\sum_{k=1}^{\infty} \frac{1}{k \cdot (k+2)}$$

1.2.3 Task 3 (Involving a proof by contradiction)

Proof the following claim and justify each of your steps:

Every convergent series is bounded.

1.2.4 Task 4 (Involving the recursive definition of a sequence)

Show that a convergent sequence of real numbers $(a_n)_{n \in \mathbb{N}}$ exists, which meets the following conditions

- $a_n = 3^{-n}$ for all even $n \in \mathbb{N}$
- $a_{n+1} = a_n + a_{n-1}$

for all $n > 1$.

Justify each of your steps.
